# Metformin Lowers Serum Cobalamin without Changing Other Markers of Cobalamin Status: A Study on Women with Polycystic Ovary Syndrome

**DOI:** 10.3390/nu5072475

**Published:** 2013-07-05

**Authors:** Eva Greibe, Birgitta Trolle, Mustafa V. Bor, Finn F. Lauszus, Ebba Nexo

**Affiliations:** 1 Department of Clinical Biochemistry, Aarhus University Hospital, Norrebrogade 44, 8000 Aarhus C, Brendstrupgaardsvej 100, 8200 Aarhus N, Denmark; E-Mails: mustabor@rm.dk (M.V.B.); e.nexo@dadlnet.dk (E.N.); 2 Department of Obstetrics and Gynecology, Aarhus University Hospital, Brendstrupgaardsvej 100, 8200 Aarhus N, Denmark; E-Mail: ditte.trolle@dadlnet.dk; 3 Department of Obstetrics and Gynecology, Herning Hospital, Gl. Landevej 61, 7400 Herning, Denmark; E-Mail: finn.friis.lauszus@vest.rm.dk

**Keywords:** vitamin B12, cobalamin, metformin, Polycystic Ovary Syndrome, haptocorrin, holotranscobalamin

## Abstract

Treatment with the anti-diabetic drug metformin is followed by a decline in plasma cobalamin, but it is unsettled whether this denotes an impaired cobalamin status. This study has explored changes in the markers of cobalamin status in women with Polycystic Ovary Syndrome treated with metformin (1.5-2.5 g per day) (*n* = 29) or placebo (*n* = 23) for six months. Serum samples were collected before and after two, four, and six months of treatment. We found serum cobalamin to decline and reach significant lower levels after six months of treatment (*p* = 0.003). Despite the decline in serum cobalamin, we observed no reductions in the physiological active part of cobalamin bound to transcobalamin (holotranscobalamin), or increase in the metabolic marker of cobalamin status, methylmalonic acid. Instead, the non-functional part of circulating cobalamin bound to haptocorrin declined (*p* = 0.0009). Our results have two implications: The data questions whether metformin treatment induces an impaired cobalamin status in PCOS patients, and further suggests that serum cobalamin is a futile marker for judging cobalamin status in metformin-treated patients.

## 1. Introduction

Metformin is an oral anti-diabetic drug known to reduce serum cobalamin (vitamin B12) concentrations in patients with type 2 diabetes mellitus [1,2,3,4,5]. For this reason, metformin is considered a risk factor for development of cobalamin deficiency, and recommendations for monitoring of patients on metformin treatment for cobalamin deficiency have been proposed [1,4,6,7]. Metformin is also used for treatment of women with Polycystic Ovary Syndrome (PCOS) [8]. A study performed in this group of patients observed a decline in plasma cobalamin after 16 weeks of metformin treatment, but showed no changes in the metabolic markers of cobalamin status, methylmalonic acid (MMA) and total homocysteine (tHcy) [9].

Cobalamin is involved in a number of key metabolic processes including red blood cell production and nervous system function, and cobalamin deficiency can lead to anemia and neurological manifestations [10,11]. In the circulation, cobalamin consists of two fractions; the fraction bound to transcobalamin (TC), holotranscobalamin (holoTC, active cobalamin), which is available to the cells; and the fraction bound to haptocorrin (HC), cobalamin-haptocorrin (Cbl-HC), whose function remains unsettled [12]. The mechanism underlying the effects of metformin on serum cobalamin concentrations remains to be elucidated; however, impaired cobalamin absorption has been proposed [4,6,13]. Recently, Obeid *et al.* (2012) found metformin treatment to improve the intracellular cobalamin metabolism in type 2 diabetic patients despite a low serum cobalamin [14]. In concordance with this, our results from an experimental study in rats show that metformin lowers serum cobalamin by increasing tissue accumulations, rather than inhibiting absorption [15].

Here we expand previous studies by analyzing the serum concentrations of total cobalamin, holoTC, totaltranscobalamin (totalTC), Cbl-HC, totalhaptocorrin (totalHC), and the metabolic marker MMA in women with PCOS before and after up to six months of treatment with metformin or placebo.

## 2. Experimental Section

### 2.1. Participants and Study Design

This study included two cohorts of women diagnosed with PCOS according to the Rotterdam criteria and treated with metformin. Cohort I came from a cross-over study on the efficacy of metformin (1.7 g per day) in obese and non-obese women with PCOS [16]. From this study, we included samples from women (*n* = 23) treated with metformin and placebo. The inclusion criteria were women (aged 18–45 years) diagnosed with PCOS with a BMI > 25 kg/m^2^, and the exclusion criteria were diabetes mellitus, elevated blood pressure, abuse of alcohol and drugs, and chronic disease. The study was approved by the Central Denmark Region Ethics Committee (no. 2166-00 and 1-10-72-540-12) and carried out from 2001 to 2002. Cohort II (*n* = 6) was diagnosed with PCOS at the Department of Obstetrics and Gynecology at Aarhus University Hospital, Denmark, from 2011 to 2012. Before inclusion, these women were scheduled to initiate treatment with metformin (1.5–2.5 g per day) by their gynecologist. The inclusion criteria age range was 18–45 years, and the exclusion criteria were treatment with either metformin or cobalamin within the past year, chronic diseases, and pregnancy. The study was approved by the Central Denmark Region Ethics Committee (no. 20100283). Our study was performed within the confines of the Helsinki Declaration, and all participants gave their informed consent before inclusion.

### 2.2. Blood Sample Collection and Storage

Non-fasting serum samples were collected at baseline (cohorts I and II), and after two (cohort I), four (cohorts I and II), and six (cohort I) months of metformin treatment. Samples were stored at −80 °C at the Department of Obstetrics and Gynecology, Herning Hospital, Denmark (cohort I) and at −25 °C the Department of Clinical Biochemistry, Aarhus University Hospital, Denmark (cohort II), until usage. In-house experiments suggest that cobalamin and its binding proteins are stable for at least 10 years when stored at −25 °C, and this is supported by others [17]. Collected samples from both cohorts were analyzed for cobalamin and related variables within four months in 2012/2013 (see below).

### 2.3. Biochemical Measurements

Total cobalamin was assayed on an automatic Cobas 6000 (Roche, Japan) system that uses alkaline hydrolysis (sodium hydroxide and dithiothreitol) for cobalamin extraction, and sodium cyanide for conversion into cyanocobalamin. In the assay, cobalamin in the sample competes with biotin-labeled cobalamin for binding to ruthenium-labeled intrinsic factor. The assay has a total imprecision of 6.6%. TotalTC was measured by an in-house sandwich ELISA with a total imprecision of 4%–6% [18]. HoloTC in serum was measured by the TC-ELISA after removal of the apoTC with cobalamin-coupled beads [19]. The total imprecision was 8% [19]. TotalHC in serum was measured by an in-house sandwich ELISA with a total imprecision of 5% [20]. Cbl-HC was calculated as total cobalamin minus holoTC. Serum MMA was measured by a LCMSMS 6490 (Agilent Technologies) with a total imprecision of 10%. The method is as described in [21], but with Multiple Reaction Monitoring instead of Single Ion Monitoring. Creatinine was measured by the Cobas 6000 (Roche, Japan) with a total imprecision of 3.9%. Blood hemoglobin and mean cell volume (MCV) were both assessed on the Sysmex XF5000 platform (Sysmex, Japan) with a total imprecision of 2% for hemoglobin and 2.2% for EMV.

### 2.4. Statistically Analysis

The D’Agostino-Pearson omnibus test was used to determine if data followed the Gaussian distribution. To test for baseline differences between the two cohorts of metformin-treated women, we used the two-tailed unpaired *t*-test or the non-parametric Mann-Whitney test when appropriate. Since no differences in serum cobalamin was found between the two cohorts at any time point, the datasets were combined and treated as one during the following statistical analysis. To compare baseline value between the combined metformin group and the placebo group, the two-tailed unpaired *t*-test or the non-parametric Mann Whitney test was used. To compare changes between baseline and a given time point for each group, we used the two-tailed paired *t*-test or the Wilcoxon signed rank test. *p*-values ≤ 0.05 were accepted as statistically significant. Data analysis was performed using the statistical software available in GraphPad Prism version 5. Data are given as median (range) if not otherwise stated.

## 3. Results

### 3.1. Characteristics of the Study Population

The group of metformin-treated women with PCOS (*n* = 29) were recruited from two different study cohorts, cohort I (*n* = 23) and cohort II (*n* = 6). The decision to merge the two cohorts was based on agreements in weight (89 (60–125) kg) and body mass index (BMI) (32 (21–46) kg/m^2^) at the time of inclusion; and more importantly the fact that none of the women took cobalamin supplements during the study. The only difference between the two cohorts was related to their age, 34 (19–44) years (cohort I) and 23 (20–37) years (cohort II). Accordingly, the demographic data for the placebo-treated group was 89 (60–119) kg (weight), 32 (21–46) kg/m^2^ (BMI) and 34 (19–44) years (age).

### 3.2. Markers of Cobalamin Status

**Figure 1:**
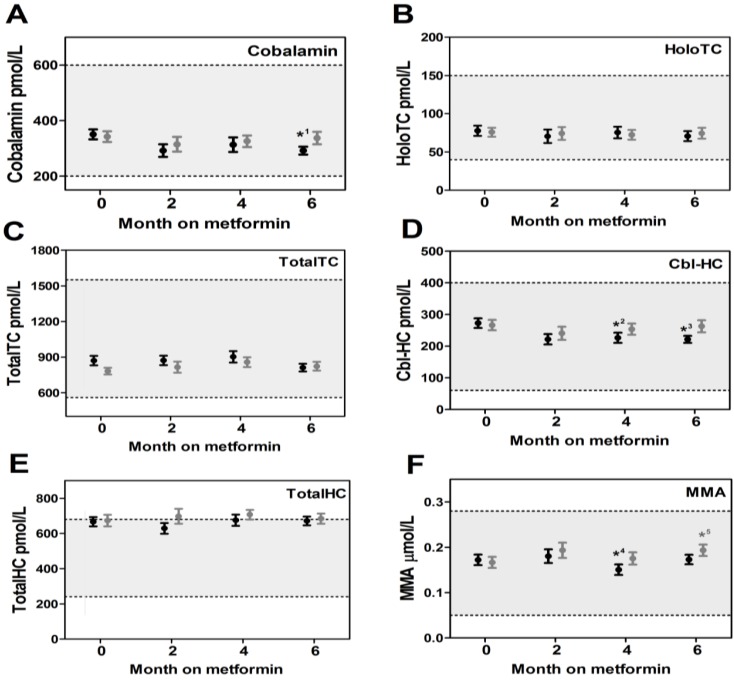
Serum markers for cobalamin status in women with Polycystic Ovary Syndrome (PCOS) on treatment with metformin or placebo. Serum cobalamin (**A**), holotranscobalamin (holoTC) (**B**), totaltranscobalamin (totalTC) (**C**), cobalamin-haptocorrin (Cbl-HC) (**D**), totalhaptocorrin (totalHC) (**E**), and methylmalonic acid (MMA) (**F**) in women with PCOS before and during treatment with metformin (black) or placebo (grey). To compare changes between baseline and a given time point, the two-tailed paired *t*-test or the Wilcoxon signed rank test was used. Due to incomplete sample material, the number of baseline paired samples varied with the time points (metformin: two (*n* = 16), four (*n* = 26), and six (*n* = 23) months; placebo: two (*n* = 16), four (*n* = 20), and six (*n* = 23) months). Results are presented as mean with SEM. Reference intervals for healthy adults [19,20,22,23] are indicated as a light grey fill between dotted lines. Reference interval for Cbl-HC was calculated from [20]. *p*-values ≤ 0.05 were considered statistical significant and are denoted with asterisks above the error bars. *^1^* p* = 0.003, *^2^* p* = 0.012, *^3^* p* = 0.0009, *^4^* p* = 0.02, *^5^* p* = 0.02.

Markers of cobalamin status were measured at baseline and after two, four, and six months of metformin or placebo treatment (Figure 1). The results (median and ranges) are also presented in Table S1. No difference between the metformin group and the placebo group was found at baseline for any of the measured parameters. In women on metformin treatment, a decline in serum cobalamin was observed after six months of treatment (*p* = 0.003). This decrease in serum cobalamin was accompanied by a decline in Cbl-HC that was significant after four months of treatment (four months, *p* = 0.012; six months, *p* = 0.0009) (Figure 1D). No overall changes in response to metformin treatment were observed in holoTC, totalTC, and totalHC (Figure 1), or in creatinine, hemoglobin, and MCV (data not shown). A small decline in MMA (*p* = 0.02) was observed after four months of metformin treatment. For the placebo group, no changes in cobalamin, its binding proteins, and the hematological parameters were observed; however a small unexplained increase in MMA (*p* = 0.02) was seen after six months of treatment.

## 4. Discussion

Our study investigates the cobalamin status during six months of metformin treatment, but provides no information on longer periods of treatment. Despite this limitation, several interesting result are obtained from this study. We confirm previous studies showing that metformin treatment decreases the levels of circulating cobalamin [1,2,3,4,5,9]. Our detailed results on the cobalamin-binding proteins indicate that the total concentration of both TC and HC remain unchanged, and that no change occurs in the active part of the circulating cobalamin, holoTC. The changes in circulating cobalamin are caused by a decrease in the fraction bound to HC. In accord with the lack of changes in holoTC, we observed no increase in the metabolic marker of cobalamin status MMA at any time point. This suggests that women with PCOS after six months of metformin treatment are sufficient in their intracellular cobalamin status and not at risk of developing cobalamin deficiency. These findings support earlier data in metformin-treated diabetic patients [14,24], and two recent studies: One study demonstrates that though metformin-treated diabetic patients have low concentrations of serum cobalamin, the intracellular cobalamin metabolism is improved [14]. Another study on rats treated with metformin reports a decline in circulating cobalamin, but an increased accumulation of cobalamin in the liver [15]. These results, taken together with our current findings, suggest that metformin-treated patients do not suffer from an impaired cobalamin status. In contrast, one may argue that total plasma cobalamin is not at useful marker for cobalamin deficiency in these patients. Preferable, cobalamin deficiency should be evaluated by measurement of holoTC and/or MMA.

We observed that the decline in serum cobalamin was accompanied by a decrease in the amount of cobalamin bound to HC, Cbl-HC. This cobalamin is inaccessible to the cells, thus provides no relevant source of cobalamin for the body. The decline in Cbl-HC supports earlier findings from a small study on ten diabetic patients [24]. We observed no change in totalHC, which indicates an increase in apoHC; the fraction of HC not saturated with cobalamin. We have previously observed the same alteration between apoHC and holoHC in pregnant women, though in this study group, totalHC declined as well [25]. At present we have no explanations to offer for findings of an altered distribution between apoHC and holoHC in relation to metformin treatment.

## 5. Conclusions

In conclusion, our data show that metformin causes a decline in serum cobalamin after six months of treatment, while no significant change occur in any of the other markers of cobalamin status. Together with previous data, our study suggests the changes in plasma cobalamin to be of no relevance for the cobalamin status of metformin-treated PCOS patients, at least for the first six months of treatment. Because of this, it is recommended not to use plasma cobalamin alone when judging an impaired cobalamin status in patients treated with metformin. Additionally, the results question whether low plasma cobalamin in metformin-treated diabetic patients causes a true or false cobalamin deficiency—an important topic that warrants future investigations.

## Supplementary Files

Supplementary File 1 Supplementary Information (DOCX, 27 KB)
