# Supplementary material for: Metformin Lowers Serum Cobalamin without Changing Other Markers of Cobalamin Status: A Study on Women with Polycystic Ovary Syndrome
Source: Nutrients. 2013 Jul 5;5(7):2475–82. doi: 10.3390/nu5072475 (PMC3738982; doi:10.3390/nu5072475)
Supplement: Supplementary File 1 — Supplementary Information (DOCX, 27 KB) [file nutrients-05-02475-s001.docx]

**Supplementary Information**

**Table S1.** Serum markers for cobalamin status in PCOS women on treatment with metformin or placebo. Serum cobalamin, holoTC, totalTC, Cbl-HC, totalHC, and MMA in women with PCOS before and during treatment with metformin (1.5–2.5 g per day) and placebo. Median and [range] are indicated. Due to incomplete samples materials, the number *n* of available results varied with each time point. For this reason, *n* is indicated. Reference intervals for healthy adults [1–4] are indicated. Reference interval for Cbl-HC was calculated from [2]. To compare changes between baseline and a given time point, the two-tailed paired *t*-test or the Wilcoxon signed rank test was used. To convert the values for cobalamin to picogram per milliliter, multiply by 1.35.

| **Plasma markers for cobalamin status PCOS-women on metformin**  **Median and Range** | | | | | |
| --- | --- | --- | --- | --- | --- |
|  | **Ref. Int.** | **Baseline** | **2 months** | **4 months** | **6 months** |
| **Cobalamin**, pmol/L  Metformin  Placebo | 200–600 | 322(145–552), *n* = 29  334(184–554), *n* = 23 | 272(169–515), *n* = 16  298(194–607), *n* = 16 | 286(142–733), *n* = 26  321(183–615), *n* = 20 | 298(183–478), *n* = 23 *  346(155–601), *n* = 23 |
| **HoloTC**, pmol/L  Metformin Placebo | 40–150 | 70(25–175), *n* = 29  72(32–141), *n* = 23 | 62(30–165), *n* = 16  67(30–165), *n* = 16 | 63(34–184), *n* = 26  75(29–134), *n* = 20 | 57(34–152), *n* = 23  67(31–150), *n* = 23 |
| **TotalTC**, pmol/L  Metformin Placebo | 560–1550 | 823(400–1435), *n* = 29  768(595–1160), *n* = 23 | 913(640–1120), *n* = 16  789(505–1225), *n* = 16 | 860(520–1495), *n* = 26  790(615–1210), *n* = 20 | 800(545–1120), *n* = 23  780(495–1250), *n* = 23 |
| **TotalHC**, pmol/L  Metformin Placebo | 240–680 | 674(369–943), *n* = 29  640(333–1195), *n* = 23 | 610(450–906), *n* = 16  707(376–1025), *n* = 16 | 700(297–939), *n* = 26  693(502–904), *n* = 20 | 657(532–1011), *n* = 23  680(449–949), *n* = 23 |
| **Cbl-HC**, pmol/L  Metformin Placebo | 103–432 | 248(120–442), *n* = 29  277(140–452), *n* = 23 | 223(128–350), *n* = 16  216(146–439), *n* = 16 | 212(126–461), *n* = 26 *****  254(144–666), *n* = 20 | 231(111–344), *n* = 23 *  267(121–492), *n* = 23 |
| **MMA**, µmol/L  Metformin Placebo | 0.1–0.3 | 0.155(0.07–0.320), *n* = 29  0.154(0.07–0.303), *n* = 23 | 0.173(0.1–0.309), *n* = 16  0.198(0.09–0.317), *n* = 16 | 0.141(0.063–0.332), *n* = 26 *****  0.170(0.092–0.317), *n* = 20 | 0.17(0.088–0.284), *n* = 23  0.188(0.9–0.317), *n* = 23 * |

* indicates *p*-values ≤ 0.05.

References

1. Nexo, E.; Christensen, A.L.; Hvas, A.M.; Petersen, T.E.; Fedosov, S.N. Quantification of
   holo-transcobalamin, a marker of vitamin B12 deficiency. *Clin. Chem.* **2002**, *48*, 561–562.
2. Morkbak, A.L.; Pedersen, J.F.; Nexo, E. Glycosylation independent measurement of the cobalamin binding protein haptocorrin. *Clin. Chem. Acta* **2005**, *356*, 184–190.
3. Nexo, E. Variation with age of reference values for P-cobalamin. *Scand. J. Haematol.* **1983**, *30*, 430–432.
4. Rasmussen, K.; Moller, J.; Lyngbak, M.; Pedersen, A.M.; Dybkjaer, L. Age- and gender-specific reference intervals for total homocysteine and methylmalonic acid in plasma before and after vitamin supplementation. *Clin. Chem.* **1996**, *42*, 630–636.
